# Prevalence and Predictors of MASLD and Fibrosis in an Urban Outpatient Setting: A Cross-Sectional Study

**DOI:** 10.3390/jcm15124533

**Published:** 2026-06-11

**Authors:** Nicolás Ortiz-López, Daniela Simian, Máximo Cattaneo, Katherine Rojas, Daniel Durán, Martina Contreras, Diego Lizama, María Fernanda Eyssautier, Camila Meza, Catalina Molina, Gerardo Jara, Jaime Poniachik

**Affiliations:** 1 Faculty of Medicine, University of Chile, Santiago 8330111, Chile; nicolas.ortiz@ug.uchile.cl (N.O.-L.); daniel.duran.a@ug.uchile.cl (D.D.); martina.contreras.m@ug.uchile.cl (M.C.); diego.lizama.r@ug.uchile.cl (D.L.); camila.meza.a@ug.uchile.cl (C.M.); catalina.molina.s@ug.uchile.cl (C.M.); gerardo.jara.a@ug.uchile.cl (G.J.); 2 Section of Gastroenterology, Department of Internal Medicine, University of Chile Clinical Hospital, Santiago 8380453, Chile; dsimian@hcuch.cl (D.S.); maximocattaneo@gmail.com (M.C.); krojas@hcuch.cl (K.R.); 3 Department of Imaging, University of Chile Clinical Hospital, Santiago 8380453, Chile; maria.eyssautier@uchile.cl

**Keywords:** diagnostic imaging, fatty liver, liver diseases, non-alcoholic fatty liver disease, prevalence

## Abstract

**Background/Objectives**: This study aims to estimate the prevalence of MASLD in a general outpatient population, describe associated metabolic risk factors, and evaluate liver fibrosis. **Methods**: We conducted a prospective, cross-sectional study at a tertiary hospital that included adults referred there for abdominal ultrasound for non-hepatic indications. Exclusion criteria were significant alcohol intake or known liver disease. Hepatic steatosis was assessed by ultrasound in all patients, and vibration-controlled transient elastography (VCTE) was performed in a subgroup. Clinical and laboratory data were collected. Comparisons used the chi-square test, Fisher’s exact test, and the Wilcoxon test, and logistic regression identified associated factors. **Results**: Hepatic steatosis was identified by ultrasound in 57.6% of the 182 patients, with most (93%) fulfilling the MASLD criteria. MASLD was diagnosed in 58.2% of patients based on ultrasound or VCTE findings of steatosis. Hepatic steatosis by ultrasound was associated with higher BMI (OR 1.30; 95% CI 1.18–1.43), hypertension (OR 1.92; 95% CI 1.04–3.53), glucose disorders (OR 3.33; 95% CI 1.60–6.92), and triglycerides (OR 1.01; 95% CI 1.00–1.03), while physical activity was protective (OR 0.86; 95% CI 0.26–0.99). Among 74 patients evaluated by VCTE, 8% had fibrosis (≥F1), which was more frequent in those with higher BMI and a number of cardiometabolic risk factors. **Conclusions**: This study reveals a high prevalence of MASLD and fibrosis among outpatients, supporting the use of abdominal ultrasound for opportunistic screening of MASLD and emphasizing the need for early risk stratification and referral.

## 1. Introduction

Metabolic dysfunction-associated steatotic liver disease (MASLD) is the most prevalent chronic liver disease (CLD) worldwide [1]. It is defined by the presence of hepatic steatosis, accompanied by at least one of the following cardiometabolic risk factors: general or central obesity, altered glucose metabolism, hypertension, or dyslipidemia, including hypertriglyceridemia or reduced high-density lipoprotein (HDL) cholesterol [2,3]. A recent meta-analysis estimated the global prevalence of MASLD at 38%, rising to 44.4% in Latin America [4]. Projections indicate a substantial global increase in incident cases over the coming decades [5]. 

In Chile, data on MASLD prevalence remain limited, and information on fibrosis burden is scarce. A population-based study conducted over a decade ago reported a hepatic steatosis prevalence of 23% [6], and a more recent retrospective analysis from a preventive medicine unit estimated MASLD prevalence at approximately 25% [7]. Both figures are lower than contemporary international estimates, raising concerns that the current national burden may be underestimated. In the context of rising obesity and metabolic syndrome nationwide [8], updated epidemiologic data are warranted.

Beyond being the leading cause of CLD, MASLD contributes substantially to hepatocellular carcinoma and liver-related mortality, emphasizing the need for early identification and risk stratification [9]. Abdominal ultrasound is the most accessible screening tool for hepatic steatosis [10], whereas vibration-controlled transient elastography (VCTE) is mainly applied for secondary risk stratification of advanced fibrosis, typically after initial screening with simple blood-based scores, rather than as a primary screening modality [11,12]. However, their combined use in outpatient populations undergoing imaging for non-hepatic indications remains unexplored in Chile.

This study aimed to estimate the current prevalence of MASLD in a general outpatient population, describe associated metabolic risk factors, and assess liver fibrosis through VCTE.

## 2. Materials and Methods

### 2.1. Study Design and Setting

We conducted an observational, cross-sectional study with prospective enrollment at the Hospital Clínico Universidad de Chile, a tertiary hospital, between May and October 2023. This manuscript follows the STROBE checklist for cross-sectional studies.

### 2.2. Participants and Sampling

Adult patients referred for abdominal ultrasound for non-hepatic indications were consecutively enrolled using a convenience sampling approach. Exclusion criteria included significant alcohol intake (>30 g/day in men, >20 g/day in women), known liver disease, or technical limitations for steatosis assessment. Clinical history, comorbidities, alcohol use, and anthropometric measurements were recorded for all participants, and recent laboratory results (≤6 months) were recorded when available.

### 2.3. Assessment of Hepatic Steatosis by Ultrasound

The ultrasound was performed by a radiologist blinded to the patient’s clinical and laboratory background during assessment of hepatic steatosis.

### 2.4. Assessment of Hepatic Steatosis and Fibrosis by Transient Elastography

All participants undergoing abdominal ultrasound were invited to undergo hepatic VCTE with FibroScan^®^ (Echosens, Paris, France); a subset completed the examination within three months based solely on patient acceptance. Examinations were performed by trained professionals and interpreted by a hepatologist. CAP (dB/m) quantified steatosis, and LSM (kPa) assessed fibrosis. Fibrosis was defined as LSM ≥ 6.2 kPa and staged according to MASLD-specific cutoffs proposed by the ALEH Working Group: F1 = 6.2–7.5, F2 = 7.6–8.7, F3 = 8.8–11.7, F4 ≥ 11.8 kPa [10].

### 2.5. Statistical Analysis

Descriptive statistics were applied. Comparisons between groups were performed using the chi-square test or Fisher’s exact test for categorical variables, as appropriate, and the Wilcoxon rank-sum test for continuous variables. Fisher’s exact test was used for analyses involving small subgroup sizes. Univariate logistic regression identified risk factors for hepatic steatosis, with *p* < 0.05 as the threshold for statistical significance. Analyses were conducted using Stata v15.0 (StataCorp LLC, College Station, TX, USA).

The sample size was calculated assuming an expected 23% prevalence of ultrasound-detected hepatic steatosis in the Chilean population [6], with a 10% absolute precision and a 95% confidence level, yielding an estimated minimum of 68 participants. To account for an anticipated 20% attrition rate, the final target sample size was increased to 85 participants.

Sankey diagrams were created to illustrate the cross-classification of steatosis by ultrasound and VCTE using the tool SankeyMATIC (https://sankeymatic.com/).

### 2.6. Ethical Considerations

This study was approved by the Ethics Committee of the Hospital Clínico Universidad de Chile (No. 040/23). Written informed consent was obtained from each participant.

## 3. Results

### 3.1. Characterization of the Patients

**Figure 1:**
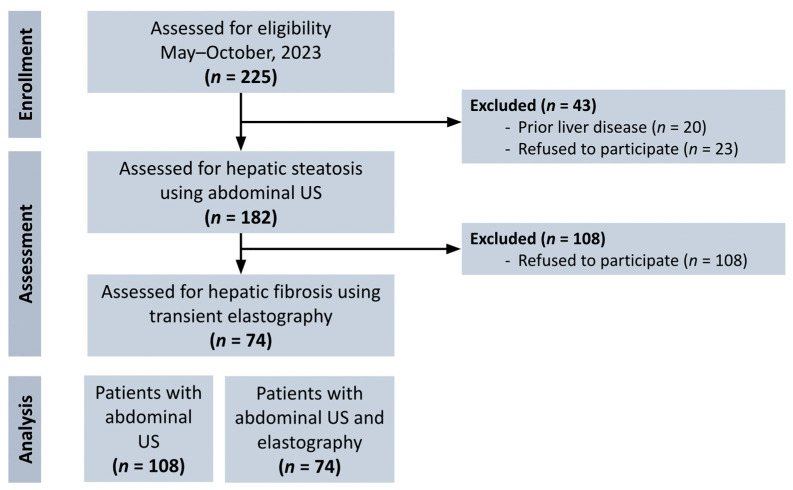
Study design and flowchart.

This study included 182 adults (19–86 years; median 55), 65% of whom were women. Ultrasound detected hepatic steatosis in 105 patients (57.6%) (Figure 1). Compared with non-steatosis patients, those with steatosis reported less physical activity and HDL levels, and had higher BMI, hypertension, and glucose-metabolism disorders. Table 1 summarizes clinical and demographic features. Univariate logistic regression (Table 2) showed positive associations of steatosis with hypertension, glucose abnormalities, and elevated triglycerides, and a negative association with physical activity.

### 3.2. Non-Invasive Characterization of Liver Fibrosis and Steatosis Grade

Regarding fibrosis characteristics, 74 patients were evaluated using VCTE. Six of them (8%) present some degree of fibrosis. Fibrosis was more frequent in patients with a higher BMI and number of cardiometabolic risk factors. No significant differences were observed in age, sex, or family history of CLD. Table 3 presents the characteristics of patients with and without fibrosis. Among the six patients with fibrosis, four were classified as F1, one as F3, and one as F4, with no cases of F2 identified.

**Figure 2:**
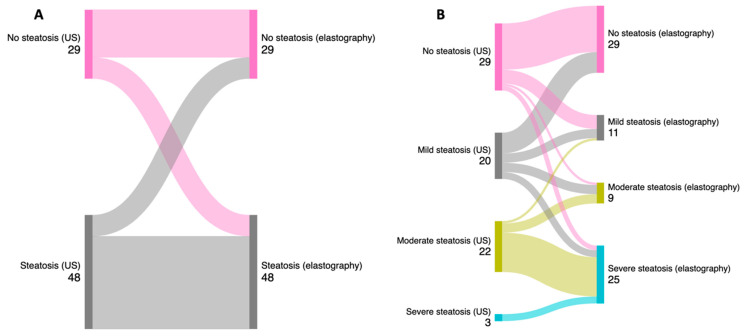
Sankey diagrams showing the cross-classification of hepatic steatosis between ultrasound and elastography. Panel (**A**) compares global categorization into no steatosis versus steatosis. Panel (**B**) displays the detailed distribution of steatosis severity based on elastography for each ultrasound category.

Cross-classification between ultrasound and VCTE revealed diagnostic discrepancies and potential reclassification between modalities. Nine (4.9%) patients without steatosis on ultrasound showed steatosis on VCTE, while others showed varying degrees of steatosis. The corresponding diagram is shown in Figure 2, and a detailed cross-tabulation appears in Appendix A.

### 3.3. Comparison of Patients with and Without MASLD

We compared the clinical and metabolic characteristics of patients with MASLD (*n* = 106) and without MASLD (*n* = 76). As shown in Table 4, MASLD patients had higher BMI, more hypertension and glucose disorders, and lower physical activity.

## 4. Discussion

In this cross-sectional study, we found a high prevalence of MASLD (58.2%) among adult outpatients undergoing abdominal ultrasound and VCTE in a tertiary center in Santiago, Chile. This rate surpasses previous national estimates and aligns with recent global trends, indicating a rising burden of MASLD. Notably, the observed prevalence more than doubled the historical 23% estimate used for sample size calculation, likely reflecting the evolving metabolic profile of the Chilean population and the systematic prospective assessment of a contemporary outpatient cohort. Our findings provide updated epidemiologic data for MASLD in Chile and highlight the value of imaging-based screening. 

Several metabolic risk factors were significantly associated with hepatic steatosis, including higher BMI, hypertension, diabetes, and elevated triglycerides. These associations align with the pathophysiological basis of MASLD, driven by insulin resistance, lipotoxicity, and low-grade inflammation [13,14]. Conversely, greater weekly physical activity was associated with a protective effect, highlighting the importance of lifestyle factors in preventing hepatic steatosis [15]. 

Ultrasound effectively detects moderate-to-severe steatosis, showing high specificity but reduced sensitivity in mild cases and in obese individuals [16]. In contrast, VCTE provides more consistent accuracy; for instance, a study reported 91.9% sensitivity and 85.7% specificity at a 247 dB/m threshold [17]. In our cohort, nine patients without steatosis on ultrasound were found to have steatosis on VCTE, predominantly in mild cases. This suggests that ultrasound alone may underestimate disease burden [18]. However, these findings support a stepwise diagnostic approach in which ultrasound serves as a frontline, cost-effective screening modality in general outpatient populations, particularly in individuals without severe obesity or advanced metabolic burden. In contrast, VCTE may be better suited for patients with multiple cardiometabolic risk factors, elevated BMI, inconclusive ultrasound findings, or those requiring fibrosis risk stratification [19]. This pragmatic approach balances accessibility with diagnostic precision in routine clinical practice.

Fibrosis represents a critical stage in the progression of MASLD and is the strongest predictor of liver-related outcomes [20,21]. In our study, fibrosis was identified in 8% of patients evaluated by VCTE (≥F1), and advanced fibrosis (≥F3) in 2.7%, similar to the 3.3% prevalence reported in the general population [22]. Although the prevalence may be underestimated due to the exclusion of patients with known liver disease, this is, to our knowledge, the first study in Chile to report fibrosis prevalence in an unselected outpatient population. Notably, fibrosis was more frequent among individuals with a higher number of cardiometabolic risk factors, consistent with recent reports linking metabolic burden to fibrosis progression in MASLD [23]. Future studies should clarify whether patients with a high cumulative metabolic risk burden may benefit from VCTE assessment beyond current screening strategies.

Our findings highlight the importance of opportunistic screening for MASLD. Abdominal ultrasound provides a valuable opportunity to detect steatosis in asymptomatic individuals. Given the silent course of early MASLD, incidental imaging findings may enable timely intervention. However, these findings are often underrecognized or poorly documented [24]. Similarly, large-scale analyses using natural language processing (NLP) have shown that steatosis, as described in imaging reports, is frequently omitted from structured diagnostic codes [25]. Emerging large language models (LLMs), such as GPT-4, have demonstrated excellent accuracy in extracting mentions of steatosis from radiology reports, offering a scalable and user-friendly strategy for detecting underreported conditions across healthcare systems [26]. In light of the high burden of MASLD observed in this outpatient population, automated extraction of steatosis mentions from ultrasound reports using NLP or LLM-based tools could facilitate systematic metabolic evaluation and timely fibrosis risk stratification.

This study has several limitations. First, other causes of hepatic steatosis beyond MASLD and alcohol-related liver disease were not systematically evaluated. Second, the single-center, geographically limited sample may restrict generalizability. Third, the use of convenience sampling could introduce selection bias. Finally, multivariable analyses were not performed to account for potential confounding factors or interactions among metabolic risk factors, and data on diet, socioeconomic status, and other social determinants were not comprehensively collected. Future research should address these factors using standardized tools.

In conclusion, this study reveals a high prevalence of MASLD among outpatients in Santiago, Chile, accompanied by frequent metabolic comorbidities and early fibrosis. These findings underscore the need for integrated clinical and policy actions addressing the social determinants of liver health through a multidimensional approach.

## Figures and Tables

**Table 1:** Characteristics of patients with or without hepatic steatosis on ultrasound.

Characteristics	Total (*n* = 182)	Steatosis (US) (*n* = 105 [57.6%])	Without Steatosis (US) (*n* = 77 [42.3%])	*p*-Value
Sex (*n*; %)				0.111
Female	118 (65)	63 (60)	55 (71)	
Male	64 (35)	42 (40)	22 (29)	
Age (median; range)	55 (19–86)	52 (21–84)	58 (19–86)	0.186
Current or former tobacco use (*n*; %)	95 (52)	56 (54)	39 (51)	0.670
Physical activity (>150 min per week) (*n*; %)	46 (25)	21 (20)	25 (32)	0.056
Physical activity (h per week) (median; range)	0 (0–14)	0 (0–8)	0 (0–14)	0.016
Cardiometabolic risk factors (*n*; %)				
Hypertension	82 (45)	55 (52)	27 (35)	0.020
Overweight or obesity	123 (75)	81 (88)	42 (59)	<0.001
Diabetes or prediabetes	49 (27)	36 (34)	13 (17)	0.009
Dyslipidemia	77 (42)	47 (45)	30 (39)	0.434
Number of cardiometabolic risk factors				<0.001
0	28 (15)	6 (6)	22 (29)	
1	49 (27)	28 (26)	22 (29)	
2	49 (27)	36 (34)	13 (17)	
3	38 (21)	23 (22)	15 (19)	
4	18 (9)	13 (13)	5 (6)	
MASLD *	106 (58)	98 (93)	8 (10)	<0.001
Family history of CLD (*n*; %)	39 (21)	20 (19)	19 (25)	0.361
BMI (median; range)	28.6 (17.4–48.3)	30.9 (21.9–48.3)	25.9 (17.4–38.7)	<0.001
Laboratory results (*n* = 52) (median; range)				
GGT	23 (6–923)	23 (6–157)	21 (14–923)	0.718
AST	26 (12–247)	27.5 (12–247)	25.5 (15–41)	0.439
ALT	27 (6–127)	29 (6–127)	21 (11–47)	0.189
Total cholesterol	180 (105–281)	177 (124–270)	183 (105–281)	0.871
HDL	47 (21–118)	40 (21–118)	56 (31–71)	0.007
LDL	104 (52–197)	100 (52–197)	111 (56–192)	0.988
Triglycerides	129 (42–412)	144 (66–412)	102 (42–189)	0.021
ALP	79 (44–566)	77 (49–132)	85 (44–566)	0.379
Fasting glucose	96 (77–214)	97 (80–171)	96 (77–214)	0.447

* MASLD is defined as hepatic steatosis by US or VCTE and at least one cardiometabolic risk factor. Abbreviations: ALP, alkaline phosphatase; ALT, alanine aminotransferase; AST, aspartate aminotransferase; BMI, body mass index; CLD, chronic liver disease; GGT, gamma-glutamyl transferase; HDL, high-density lipoprotein; LDL, low-density lipoprotein; TG, triglycerides; US, ultrasound.

**Table 2:** Univariate logistic regression of factors associated with the presence of steatosis assessed by abdominal ultrasound.

Characteristics	OR	IC95%	*p*-Value
Physical activity (>150 min per week)	0.52	0.26–1.02	0.058
Physical activity (h per week)	0.86	0.76–0.99	0.038
BMI	1.30	1.18–1.43	<0.001
Hypertension	1.92	1.04–3.53	0.036
Diabetes or prediabetes	3.33	1.60–6.92	0.001
HDL	0.97	0.93–1.01	0.200
Triglycerides	1.01	1.00–1.03	0.029

Abbreviations: BMI, body mass index; HDL, high-density lipoprotein.

**Table 3:** Comparison of sociodemographic and clinical variables between patients with and without liver fibrosis assessed by hepatic elastography.

Characteristics	Fibrosis (kPa ≥ 6.2) (*n* = 6 [8%])	Without Fibrosis (kPa < 6.2) (*n* = 68 [92%)]	*p*-Value
Sex (*n*; %)			0.109
Female	2 (33)	46 (68)	
Male	4 (67)	22 (32)	
Age (median; range)	50 (39–69)	55 (21–82)	0.526
Current or former tobacco use (*n*; %)	5 (83)	37 (55)	0.186
Physical activity (>150 min per week) (*n*; %)	1 (17)	18 (26)	0.513
Physical activity (h per week) (median; range)	0 (0–1)	0 (0–8)	0.180
Cardiometabolic risk factors (*n*; %)			
Hypertension	4 (67)	32 (47)	0.311
Overweight or obesity	5 (83)	55 (85)	0.650
Diabetes or prediabetes	4 (67)	24 (35)	0.141
Dyslipidemia	4 (67)	36 (53)	0.418
Number of cardiometabolic risk factors			0.044
0	1 (17)	4 (6)	
1	0 (0)	15 (22)	
2	1 (17)	22 (32)	
3	1 (17)	20 (29)	
4	3 (50)	7 (10)	
MASLD *	5 (83)	46 (68)	0.389
Family history of CLD (*n*; %)	2 (33)	16 (24)	0.454
BMI (median; range)	40 (22.4–48.3)	29 (20.1–47.8)	0.027
CAP (median; range)	336 (144–400)	260 (148–384)	0.219
kPa (median; range)	8.45 (6.2–75)	4.3 (2.8–6.1)	<0.001

* MASLD is defined as hepatic steatosis by US or VCTE and at least one cardiometabolic risk factor. Abbreviations: BMI, body mass index; CAP, controlled attenuation parameter; CLD, chronic liver disease; kPa, kilopascals; MASLD, metabolic dysfunction–associated steatotic liver disease.

**Table 4:** Comparison of sociodemographic and clinical variables between patients with and without MASLD.

Characteristics	MASLD (*n* = 106 [58.2%])	Without MASLD (*n* = 76 [41.7%])	*p*-Value
Sex (*n*; %)			0.072
Female	63 (59)	55 (72)	
Male	43 (41)	21 (27)	
Age (median; range)	54 (21–84)	56 (19–86)	0.730
Current or former tobacco use (*n*; %)	57 (54)	38 (50)	0.569
Physical activity (*n*; %)	21 (20)	25 (33)	0.045
Physical activity (h per week) (median; range)	0 (0–8)	0 (0–14)	0.008
Cardiometabolic risk factors (*n*; %)			
Hypertension	60 (57)	22 (29)	<0.001
Overweight or obesity	87 (90)	36 (55)	<0.001
Diabetes or prediabetes	38 (36)	11 (14)	0.001
Dyslipidemia	53 (50)	24 (32)	0.013
Number of cardiometabolic risk factors			<0.001
0	0 (0)	28 (27)	
1	28 (26)	21 (28)	
2	39 (37)	10 (13)	
3	24 (23)	14 (18)	
4	15 (14)	3 (4)	
Family history of CLD (*n*; %)	18 (17)	21 (28)	0.084
BMI (median; range)	30.8 (21.9–48.3)	25.6 (17.4–38.7)	<0.001
CAP (median; range)	279 (144–400)	215 (178–307)	<0.001
kPa (median; range)	4.5 (2.8–75)	4.3 (3–10.8)	0.269

Abbreviations: BMI, body mass index; CAP, controlled attenuation parameter; CLD, chronic liver disease; kPa, kilopascals; MASLD, metabolic dysfunction–associated steatotic liver disease.

## Data Availability

The data presented in this study are available upon request from the corresponding author. The data are not publicly available due to ethical reasons.

## Abbreviations

The following abbreviations are used in this manuscript:

ALT	alanine aminotransferase
ALP	alkaline phosphatase
AST	aspartate aminotransferase
BMI	body mass index
CAP	controlled attenuation parameter
CLD	chronic liver disease
GGT	gamma-glutamyl transferase
HDL	high-density lipoprotein
kPa	kilopascal
LDL	low-density lipoprotein
MASLD	metabolic dysfunction-associated steatotic liver disease
STROBE	Strengthening the Reporting of Observational Studies in Epidemiology
TG	triglycerides
US	ultrasound
VCTE	vibration-controlled transient elastography

## Appendix A

**Table A1:** Cross-classification of hepatic steatosis severity by ultrasound (US) and vibration-controlled transient elastography (VCTE). Rows represent US-based categories; columns represent VCTE-based steatosis grading.

	No Steatosis (VCTE)	Mild Steatosis (VCTE)	Moderate Steatosis (VCTE)	Severe Steatosis (VCTE)
No steatosis (US)	20	6	1	2
Mild steatosis (US)	9	4	4	3
Moderate steatosis (US)	0	1	4	17
Severe steatosis (US)	0	0	0	3
